# FK506-Binding Protein 22 from a Psychrophilic Bacterium, a Cold Shock-Inducible Peptidyl Prolyl Isomerase with the Ability to Assist in Protein Folding

**DOI:** 10.3390/ijms12085261

**Published:** 2011-08-17

**Authors:** Cahyo Budiman, Yuichi Koga, Kazufumi Takano, Shigenori Kanaya

**Affiliations:** 1 Department of Material and Life Science, Osaka University, 2-1 Yamadaoka, Suita, Osaka 565-0871, Japan; E-Mails: cahyo@bio.mls.eng.osaka-u.ac.jp (C.B.); kogay@mls.eng.osaka-u.ac.jp (Y.K.); kanaya@mls.eng.osaka-u.ac.jp (S.K.); 2 Graduate School of Life and Environmental Sciences, Kyoto Prefectural University, 1-5 Hangi-Cho, Shimogamo, Sakyo-ku, Kyoto 606-8522, Japan

**Keywords:** cold adaptation, peptidyl prolyl *cis-trans* isomerases (PPIases), *Shewanella* sp. SIB1, FKBP22, folding

## Abstract

Adaptation of microorganisms to low temperatures remains to be fully elucidated. It has been previously reported that peptidyl prolyl *cis-trans* isomerases (PPIases) are involved in cold adaptation of various microorganisms whether they are hyperthermophiles, mesophiles or phsycrophiles. The rate of *cis-trans* isomerization at low temperatures is much slower than that at higher temperatures and may cause problems in protein folding. However, the mechanisms by which PPIases are involved in cold adaptation remain unclear. Here we used FK506-binding protein 22, a cold shock protein from the psychrophilic bacterium *Shewanella* sp. SIB1 (SIB1 FKBP22) as a model protein to decipher the involvement of PPIases in cold adaptation. SIB1 FKBP22 is homodimer that assumes a V-shaped structure based on a tertiary model. Each monomer consists of an N-domain responsible for dimerization and a C-catalytic domain. SIB1 FKBP22 is a typical cold-adapted enzyme as indicated by the increase of catalytic efficiency at low temperatures, the downward shift in optimal temperature of activity and the reduction in the conformational stability. SIB1 FKBP22 is considered as foldase and chaperone based on its ability to catalyze refolding of a *cis-*proline containing protein and bind to a folding intermediate protein, respectively. The foldase and chaperone activites of SIB1 FKBP22 are thought to be important for cold adaptation of *Shewanella* sp. SIB1. These activities are also employed by other PPIases for being involved in cold adaptation of various microorganisms. Despite other biological roles of PPIases, we proposed that foldase and chaperone activities of PPIases are the main requirement for overcoming the cold-stress problem in microorganisms due to folding of proteins.

## 1. Introduction

Temperature has been deemed one of the most important factors for growth and modulates selection and distribution of microorganisms throughout the Earth. Microorganisms can be classified according to the range of temperatures in which they can grow: hyperthermophiles (temperature range of 80–115 °C, with optimum growth at >80 °C), thermophiles (40–110 °C, optimum ∼60 °C), mesophiles (10–50 °C, optimum ∼37 °C), psychrotrophs (0–30 °C, optimum ∼22–25 °C) and psychrophiles (0–20 °C, optimum ≤15 °C) [1].

Adaptation of microorganisms that live in extreme temperatures (either high or low) is gaining particular interest among scientists, due to the importance of understanding the molecular basis of adaptation and biotechnological applications [1]. In contrast to thermophilic and/or hyperthermophilic microorganisms, only a few studies have been conducted so far to address the adaptation to low temperatures (cold adaptation). This might be due to commercial reasons in which thermostable microorganisms or enzymes are highly valuable for biotechnological applications [2,3]. However, some cold-adapted enzymes were reported to be promising and have recently been widely applied in biotechnology [4–8]. Therefore, understanding the adaptation of microorganisms to low temperature, including the machinery enzymes, is also important for industrial purpose. The information may be also ecologically important since approximately 70% of the earth’s biosphere has a constant temperature of 4–5 °C [4,9] the reason why it is populated predominantly by psychrophilic and psychrotropic bacteria. It is noted also that some pathogenic microorganisms survive in low temperature, therefore the insight on this mechanism would be useful for controlling pathogens.

Once microorganisms are exposed to low temperatures they may face a series of problems that affect their growth. Primarily, problems encountered at low temperatures are associated with membrane viscosity and permeability, synthesis of macromolecules (e.g., replication, transcription and translation), slower metabolic rates due to lower kinetics of enzymatic reactions, and the ability to sense and transmit temperature signals to the regulatory networks of cells for effective responses [1].

Protein folding can be given as an example of problems associated with the synthesis of macromolecules and a slower metabolic rate. Folding of proteins, especially for *cis-*proline containing proteins, is suggested to be a rate-limiting step for bacterial growth in cold environments [10]. The conformations of folded proteins are usually compatible with only one isomer of a peptide bond, in which the *trans* form is preferred to the *cis* form due to energetic reasons [11]. However, in the case of peptide bonds preceding proline residues (Xaa-Pro), some of them correctly form *cis* peptide bonds in folded proteins [12]. Consequently, interconversion of *cis-trans* Xaa-Pro peptide bonds take place during folding processes and are termed as peptidyl prolyl *cis-trans* isomerizations.

**Figure 1.**
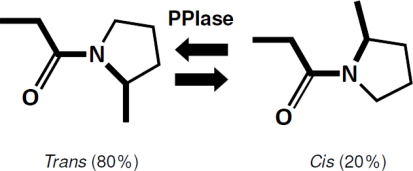
The *cis–trans* isomerization reaction catalyzed by PPIases. In a denatured state of proteins, all peptide bonds assume the energetically stable *trans* conformation. Exceptions include the peptide bond preceding the proline residue (Xaa-Pro). This bond exists in an equilibrium state, in which 80% of this bond assumes a *trans* conformation and 20% assumes a *cis* conformation. PPIase does not change this equilibrium state, but equally accelerates both rates from *trans* to *cis* and from *cis* to *trans*. For proteins containing *cis*-proline residues in a native state, *cis-trans* isomerization of the proline residue has been reported to be a rate-limiting step of protein folding.

However, the kinetics of *cis-trans* isomerization is intrinsically slow because it involves the rotation about a partial double bond. Isomerization is much slower at low temperatures, as are other chemical reactions, since it is thermodynamically linked [11]. Therefore, *cis-trans* isomerization is regarded as a rate-limiting step of protein folding. Peptidyl prolyl *cis-trans* isomerases (PPIases; EC 5.2.1.8) catalyze *cis-trans* isomerization of Xaa-Pro peptide bonds (Figure 1) and assist proteins to achieve correct native conformations. Due to this ability, PPIases are considered to be foldases, together with protein disulfide isomerases (PDI) [13,14]. Currently, at least three structurally unrelated families of PPIase have been indentified: FK506-binding proteins (FKBP), cyclophilins, and parvulins [15,16].

Psychrophilic and psychrothrophic bacteria produce a cold shock response, which is defined as the sum of cellular reactions to overcome the problems associated with decreases in temperature [1]. Cold shock responses are indicated by the expression of cold shock proteins (CSPs) or class 1 proteins, which are involved in the adaptation of microorganisms to low-temperature environments. Foldases and chaperones have been classified as CSPs, which are important for overcoming a series of problems in protein folding at low temperatures, including slow *cis-trans* isomerization [1].

Despite PPIases being clearly classified as foldases, multidomain PPIases have also been reported to exhibit chaperone functions as indicated by their ability to bind incompletely folded proteins and prevent aggregation [17–23]. In addition, Wang and Tsou [13] noted that foldases may also act as chaperones and vice versa. Therefore, the dual functions of PPIases would be an interesting topic for in-depth study in relation to their involvement in cold-adaptation.

However, to our knowledge, studies pertaining to this issue are limited. Despite some PPIases from cold-adapted organisms have been reported and linked to cold adaptation [10,24,25], these reports are based on proteomic analysis without further structural and functional studies. Our group has been extensively studying FKBP22 from the psychrotrophic bacterium, *Shewanella* sp. SIB1 (SIB1 FKBP22). Expression of SIB1 FKBP22 was significantly increased when *Shewanella* sp. SIB1 was grown at low temperatures, indicating that it is involved in cold-adaptation [26]. SIB1 FKBP22 is thought to have dual functions, since it exhibits PPIase activity (foldase) and is able to bind to a folding intermediate protein (chaperone). This review focuses on structural and functional studies of SIB1 FKBP22 and its possible involvement in cold adaptation through its dual function as a foldase and a chaperone.

## 2. *Shewanella* sp. SIB1 as a Psychrophilic Bacterium

**Figure 2.**
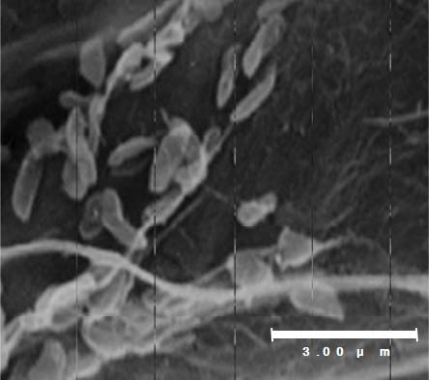
Scanning electron micrograph of *Shewanella* sp. SIB1. This figure was reproduced and modified from Kato *et al.* [27].

*Shewanella* sp. SIB1 was previously isolated from reservoir water at the Shibugaki petroleum processing plant (Teikoku Oil, Niigata, Japan). Physiological and morphological characterizations were examined using an API 20NE Kit (bioMérieux, Durham, NS, USA) and scanning electron microscopy, respectively [27]. Physiologically, strain SIB1 is able to assimilate glucose, d-mannitol, maltose and malic acid. In addition, this strain possesses cytochrome oxidase and β-glucosidase enzymes and exhibits protease activity as indicated by its ability to degrade gelatin substrates. Further biochemical characterization has shown that the SIB1 strain is consistently able to degrade a catechol (a hydroxylated aromatic hydrocarbon), although in small amounts. On the contrary, the SIB1 strain cannot degrade two other hydrocarbon compounds, namely alkenes (aliphatic carbon) and naphtalene (an aromatic hydrocarbon). Morphologically, strain SIB1 is rod-shaped with a diameter of 0.3 μm and a length of 1.0–1.3 μm (Figure 2). The 16S RNA gene of strain SIB1 shows a 99.9% homology to the 16S RNA gene of psychrotrophic *Shewanella* sp. AC10 [28], which obviously classify strain SIB1 in the genus *Shewanella*.

**Figure 3.**
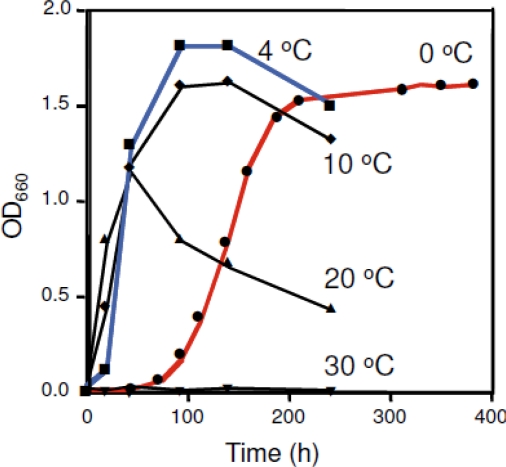
Effect of temperature on the growth of *Shewanella sp.* SIB1. Cells were grown at 0 (closed circle), 4 (closed square), 10 (closed diamond), 20 (closed triangle), and 30 °C (upside-down-closed triangle) on the same medium that was used for isolation. This figure was reproduced and modified from Kato *et al.* [27].

Growth temperature profiles of strain SIB1 have been identified [27]. Results show that the SIB1 strain does not grow at temperatures above 30 °C, while the maximum growth rate was at 20 °C. However, maximum cell density of this strain is reached at 4 °C (Figure 3). This consideration prompted us to confidently classify this strain as a psychrophile (previously classified as psychotrophic bacterium [27]). *Shewanella* sp. SIB1 is an appropriate model to decipher the mechanisms for cold-adaptation. To achieve this goal, we have studied several proteins isolated from *Shewanella* sp. SIB1, including RNase HI [29], FKBP22 [26], alkaline phosphatase [8], RNase HII [30] and CutA [31]. However, this review focuses on SIB1 FKBP22 since, among the proteins mentioned above, only this protein is involved in cold shock responses of strain SIB1 and therefore is thought to be important for cold adaptation [26].

## 3. SIB1 FKBP22 as a Cold Shock Protein

Responses of psychrotrophic and psychrophilic bacteria to low temperatures are characterized by changes in their profile of cellular contents. Some proteins that are associated with adaptation to low temperatures are highly expressed. Hence, we have investigated proteins that are important for cold adaptation of *Shewanella* sp. SIB1 by employing two-dimensional electrophoresis [26]. As a part of proteome analysis, 2D-PAGE has been widely used in order to identify putative proteins/enzymes involved in cold adaptation of various microorganisms [24,32–35].

**Figure 4.**
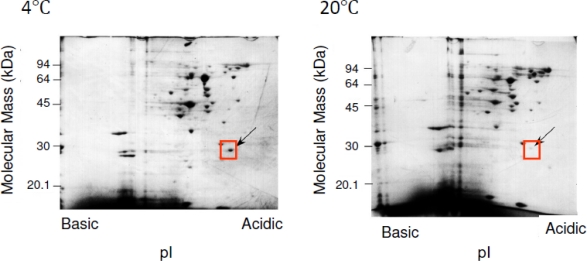
2D-PAGE analysis of the proteins extracted from SIB1 cells. Soluble proteins extracted from SIB1 cells grown at 4 and 20 °C were applied to 2D-PAGE. Slab gels were stained with Coomassie Brilliant Blue. Boxes and arrows indicate the position of P28. These figures were reproduced and modified from Suzuki *et al.* [26].

The 2D-PAGE maps were obtained from extracted soluble fractions of *Shewanella* sp. SIB1, which were grown at 4 and 20 °C. By comparing the intensity of spots in the gel at these two temperatures, we found that the cellular content of several proteins were greatly increased at 4 °C as compared to 20 °C (Figure 4). One of these proteins is referred to as P28, which has an apparent mass of 28 kDa and an isoelectric point of 4.0. Suzuki *et al.* [26] reported that when *Shewanella* sp. SIB1 is grown at 10 and 0 °C, the cellular content of P28 is increased as compared to those grown at 20 °C. However, this protein did not significantly increase at 15 °C. Collectively, P28 is thought to be greatly increased when *Shewanella* sp. SIB1 is grown at temperatures below 10 °C. This evidence prompted us to classify P28 as a cold shock protein of *Shewanella* sp. SIB1 which might be involved in cold adaptation.

Further analysis indicates that P28 shares 56% amino acid sequence identity to *Escherichia coli* FKBP22 (accession no. AAC77164) [36], 43% to *E. coli* FkpA (accession no. AAC76372) [37] and 41% to *Legionella pneumophila* MIP (accession no. S42595) [38]. These proteins are members of the PPIase family which exhibit activity towards peptide (PPIase_pep_) and protein (PPIase_pro_) substrates. Electrospray ionization mass spectrometry (ESI-MS) analysis revealed that the molecular mass of P28 was 23.9 kDa, which is comparable to that calculated from the amino acid sequence. According to the aforementioned data, P28 is defined as SIB1 FKBP22, which indicates that this protein is a member of FKBP from SIB1 with a molecular mass of approximately 22 kDa.

Oligomeric state analysis by sedimentation equilibrium analytical ultracentrifugation indicated that SIB1 FKBP22 assumes a homodimeric protein of 48 kDa, about twice as large as those calculated [26]. *L. pneumophila* MIP, *E. coli* FkpA and *E. coli* FKBP22 were also reported to be homodimeric proteins [36–38]. Therefore, SIB1 FKBP22, as well as *E. coli* FkpA, was classified to the (MIP)-like FKBP protein subfamily. However, when gel filtration was employed to analyze the oligomeric state of SIB1 FKBP22, the deduced molecular mass was 3–4 times larger than calculated, indicating that SIB1 FKBP22 forms a trimeric or tetrameric structure. This discrepancy might be caused by the shape of SIB1 FKBP22, since it is apparently a non globular protein that migrates through the gel filtration column faster than other globular proteins that were used for the calibration of molecular mass.

**Figure 5.**
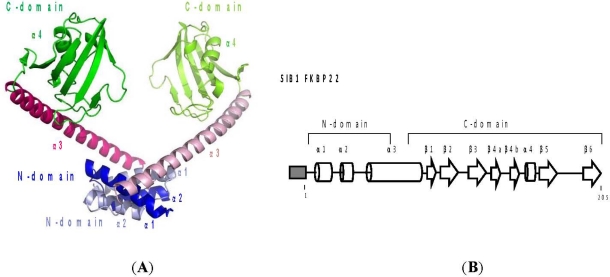
(**A**) Three-dimensional structure model of SIB1 FKBP22. One monomer is dark-colored, while the other is light-colored. The N- and C-domains and α1–4 helices are indicated; (**B**) Schematic representations of the primary structure of SIB1 FKBP22. A His-tag attached to the N-termini of the proteins is represented by the shaded box. The α-helices and β-strands are represented by cylinders and arrows, respectively. These secondary structures are arranged based on tertiary models of SIB1 FKBP22. This figure was reproduced and modified from Suzuki *et al.* [39] and Budiman *et al.* [40].

Due to the lack of a crystal structure image of SIB1 FKBP22, a three-dimensional model of this protein has been constructed based on the crystal structure of *L. pneumophila* MIP [39]. Based on this model, these proteins are predicted to assume a non-globular V-shaped homodimeric structure, in which two monomers interact with each other at their N-domains (Figure 5). Each monomer assumes a dumbbell-like structure, in which the N-domain, consisting of α1 and α2 helices, and the C-domain, consisting of six β strands (β1–β6) and an α4 helix, are linked by an α3 helix, which is 40 residues long. The C-domains, which are located at both ends of a V-shaped structure, face each other across the cleft of this structure. The interface of two monomers, which is located at the bottom of the V-shaped structure, is stabilized by the hydrophobic interactions between the α1 helix of one monomer and the α2 helix of the other.

## 4. Characterization of the Activity and Stability of SIB1 FKBP22 as a Cold-Adapted Enzyme

It is important to confirm whether SIB1 FKBP22 is a typical cold-adapted enzyme. In order to be involved in cold adaptation, a protein must adapt to low temperatures and remain fully functional. For this purpose, we have analyzed PPIase activity of SIB1 FKBP22 at various temperatures and determined its relationship to the stability of the protein.

### 4.1. PPIase Activity of SIB1 FKBP22

The PPIase activity of SIB1 FKBP22 on peptide substrates (PPIase_pep_ activity) has been examined. *N-*succinyl-Ala-Leu-Pro-Phe-*p*-nitroanilide was used as substrate since it was identified as the best substrate for FKBP family members [26]. This substrate mimics the internal peptidyl-prolyl moiety of proteins containing proline.

**Figure 6.**
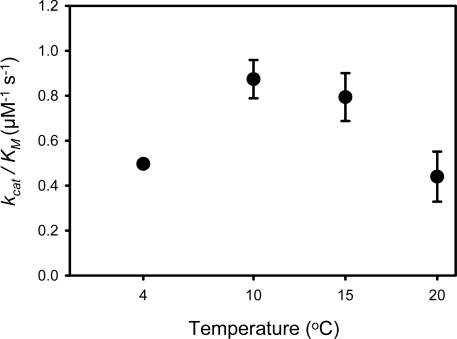
The PPIase_pep_ activity of SIB1 FKBP22 was determined by a protease coupling assay at the temperatures indicated using N-succinyl-Ala-Leu-Pro-Phe-p-nitroanilide as a substrate. The experiment was carried out in duplicate. Each plot represents the average value and standard errors of the mean are shown. This figure was reproduced and modified from Suzuki *et al.* [39].

The temperature dependence of the PPIase_pep_ activity of SIB1 FKBP22 showed that this protein is apparently exhibited catalytic efficiency at an apparent optimum temperature of 10 °C (0.87 μM^−1^ s^−1^) (Figure 6) [26], which is lower, but comparable, either to that of its mesophilic counterpart, *E. coli* FKBP22 (1.33 μM^−1^ s^−1^) [36] or human FKBP12 (1.2 μM^−1^ s^−1^) [41] when measured at 10 °C. This value is comparable to that found at 15 °C; however, it was significantly decreased at temperatures higher than 25 °C. In contrast, PPIase_pep_ activity of *E. coli* FKBP22 increased as the reaction temperature increased from 4 to 25 °C [26].

It has been previously reported that SIB1 FKBP22 is much less stable than *E. coli* FKBP22 [26]. However, its apparent optimal temperature for activity is significantly less than that of *E. coli* FKBP22. This observation follows common consensus on cold-adapted enzymes, which are specified by increases in catalytic efficiency at low temperatures, the downward shift in optimal temperatures for activity and reductions in conformational stability [9]. Therefore, SIB1 FKBP22 can be defined as a cold-adapted enzyme.

**Figure 7.**
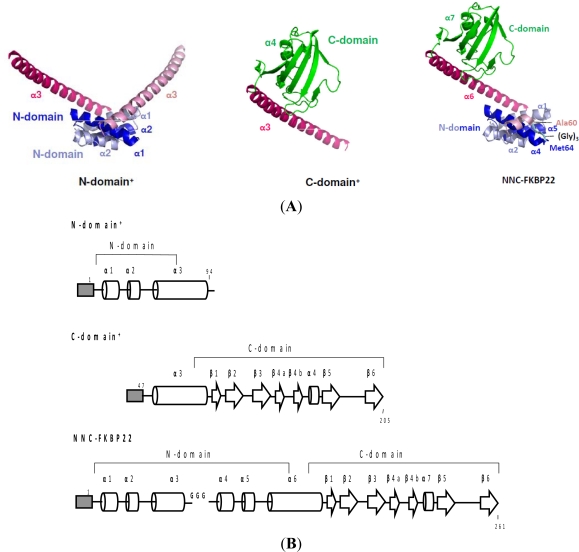
(**A**) Three dimensional structure models of SIB1 FKBP22 derivatives: N-domain^+^, C-domain^+^, and NNC-FKBP22. These models were generated from Figure 5A. For the N- and C-domain structures, α1–4 helices are shown. For the NNC-FKBP22 structure, the corresponding domains, helices, and side chains of the amino acid residues are indicated. A loop consisting of three glycine residues, which connects Ala60 and Met64 (corresponding to Met8 of SIB1 FKBP22), is schematically shown in cyan. The corresponding α1–4 helices of the second monomer in NNC-FKBP22 are ordered as α4–7 helices since the second monomer is covalently linked to the N-domain of the first monomer; (**B**) Schematic representations of the primary structures of SIB1 FKBP22 derivatives: N-domain^+^, C-domain^+^, and NNC-FKBP22. A His-tag attached to the N-termini of the proteins is represented by a shaded box. The α-helices and β-strands are represented by cylinders and arrows, respectively. These secondary structures are arranged based on tertiary models of all of SIB1 FKBP22 derivatives. These figures were reproduced and modified from Suzuki *et al.* [39].

To fully exhibit PPIase_pep_ activity, SIB1 FKBP22 does not require an intact molecule. This has been confirmed by our works on SIB1 FKBP22 derivative proteins: N-domain^+^, C-domain^+^, and NNC-FKBP22. The N- and C-domains^+^ are SIB1 FKBP22 derivatives that lack the C- and N-domains of SIB1 FKBP22, respectively, that are constructed to clarify roles of each domain for SIB1 FKBP22 [39]. NNC-FKBP22 is an engineered monomeric protein in which the N-domain is tandemly repeated throughout a flexible linker. NNC-FKBP22, which is unable to form a V-shaped structure, was constructed to clarify the role of the V-shaped structure of SIB1 FKBP22 [41]. For clarity, the three-dimensional structure models and a schematic representation of the primary structure of these SIB1 FKBP22’s derivatives are shown in Figure 7. Three dimensional models show that C-domain^+^ and NNC-FKBP22 are monomers, while N-domain^+^ is a dimer. These models are in good agreement with oligomeric states analysis conducted by using sedimentation equilibrium analytical ultracentrifugation [39,40].

**Figure 8.**
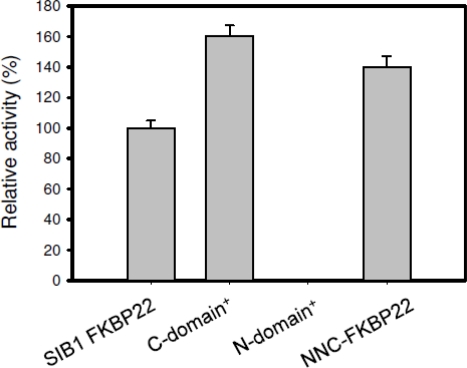
PPIase_pep_ activities of SIB1 FKBP22 and its derivatives measured at 10 °C. Relative PPIase_pep_ activities of the SIB1 FKBP22 derivatives, which are calculated by dividing the *k*_cat_/*K*_m_ value of the SIB1 FKBP22 derivatives by that of the wild-type protein (SIB1 FKBP22), are shown. The PPIase_pep_ activity was determined by a protease coupling assay using Suc-ALPF-*p*NA as a substrate. The *k*_cat_/*K*_m_ value of WT was 0.87 μM^−1^ s^−1^. This figure was reproduced and modified from Suzuki *et al.* [39].

PPIase_pep_ activities of SIB1 FKBP22 derivatives are shown in Figure 8. NNC-FKBP22 and the C-domain^+^, which are monomers, exhibited higher, but comparable, PPIase_pep_ activities to that of SIB1 FKBP22. The temperature-dependent activities of these proteins were also identical to that of SIB1 FKBP22 [37,40]. The C-domain^+^ and NNC-FKBP22 exhibited the highest activity at 10 °C and decreased at temperatures higher than 20 °C. These results indicate that C-domain alone is sufficient to bind and catalyze the isomerization reaction on a peptide substrate. These results also suggest that a V-shaped structure is not conducive to binding a peptide substrate. In this structure, the freedom of each catalytic domain is probably restricted and therefore the probability of this domain connecting with the substrate decreases. In contrast, the monomeric structure increases the freedom of the catalytic domain and therefore the chance of this domain connecting with the substrate also increases [40].

### 4.2. Stability of SIB1 FKBP22

**Figure 9.**
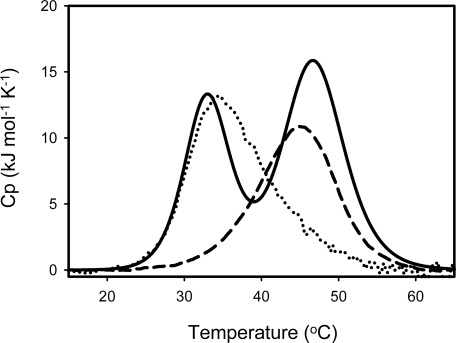
DSC curves of SIB1 FKBP22 (solid line), C-domain^+^ (dot line) and N-domain^+^ (broken line). The curves were measured at a scan rate of 1 °C min^−1^. Proteins were dissolved in 20 mM sodium phosphate (pH 8.0) in at approximate final concentration of 1 mg mL^−1^. This figure was reproduced from Suzuki *et al.* [39].

The stability of SIB1 FKBP22 has been examined by differential scanning calorimetry (DSC) [39]. Thermal unfolding of these proteins was highly reversible as indicated by repeated thermal scans to reproduce DSC curves. The denaturation curve of SIB1 FKBP22 clearly showed two well separated transitions (Figure 9). Deconvolution of the thermogram, according to a non-two-state denaturation model, resulted in melting temperature (*T*_m_) values of 32.5 °C and 46.6 °C for these transitions. Upon examination of the stabilities of the C- and N-domain^+^ (Table 1), these *T*_m_ values were nearly equal to those of the C-domain^+^ (35.6 °C) and N-domain^+^ (44.4 °C), suggesting that the thermal unfolding transitions of SIB1 FKBP22 at lower and higher temperatures represent those of its C-domain and N-domain, respectively. This result also suggested that the C-domain is less stable than the N-domain.

### 4.3. Relationship between Activity and Stability of SIB1 FKBP22

DSC data showed that unfolding of SIB1 FKBP22 initiates at a temperature higher than 25 °C. Interestingly, thermal unfolding of the C-catalytic domain is also initiated at temperatures greater than 25 °C (data not shown) [39]. Nevertheless, SIB1 FKBP22 and the C-domain^+^ exhibited maximal PPIase_pep_ activity at 10 °C, while their activities are greatly reduced at 20 °C. Meanwhile, there is no significant difference in the CD spectrum of SIB1 FKB22 at 10 and 20 °C [39], suggesting that the conformation of this protein is not significantly influenced by a temperature shift from 10 to 20 °C. However, subtle conformational changes of the active site may occur at temperatures higher than 20 °C which can promote a loss in some of the interactions between the enzyme and the substrate, leading to a decrease in the concentration of the enzyme-substrate complex, and therefore may cause a significant reduction in enzymatic activity.

Cold-adapted α-amylase and family 8 xylanase from an Antarctic bacterium also showed large differences in temperatures for enzymatic inactivation and structural unfolding [42,43]. The apparent optimal temperatures for enzymatic activities of these proteins are much lower than the temperatures at which any significant conformational event occurred. Different results have been observed for their mesophilic and thermophilic counterparts in which the optimal activation temperatures are closely correlated to the temperatures influencing structural transitions.

Based on the aforementioned observations, it is likely that cold adapted enzymes are characterized by large differences in the temperatures for enzymatic inactivation and structural unfolding. D’amico *et al.* [42] proposed that this difference is caused by a cold-adaptation strategy called “localized flexibility”. Although an increase of flexibility around the active site increases *k*_cat_ by reducing the energy cost of conformational change during the catalytic reaction, it should increase *K*_m_ concurrently. By restricting the increase of flexibility within small regions, cold adapted enzymes prevent unfavorable increases in *K*_m_ [44]. SIB1 FKBP22 probably adopts a similar strategy for cold adaptation.

## 5. Involvement of SIB1 FKBP22 in Protein Folding

The role of SIB1 FKBP22 in protein folding at low temperatures remains to be clarified. Two groups of proteins, chaperones and foldases, have been recognized as groups of accessory proteins that assist the maturation of nascent polypeptides into functional proteins within cells [13]. Chaperones are defined as a group of proteins that assist correct folding without covalent changes. They recognize and selectively bind nonnative proteins to prevent aggregation. In contrast, foldases catalyze necessary covalent reactions directly involved in protein folding. The foldases, therefore, differ from chaperones due to their involvement in covalent changes. Only two families of foldases have been characterized thus far: namely PDI and PPIases.

Wang and Tsou [13] provided interesting evidence that in some cases, foldases may act as chaperones. Likewise, chaperones may exhibit catalytic activity that is involved in folding protein. Therefore, a protein may act as foldase and chaperone to assist protein folding. In this respect, SIB1 FKBP22 may behave as a foldase and chaperone to overcome problems in protein folding at low temperatures as faced by *Shewanella* sp. SIB1.

### 5.1. SIB1 FKBP22 as a Foldase

SIB1 FKBP22 as a foldase is evidenced through its ability to accelerate folding of RNase T_1_ [26]. RNase T_1_ has been widely used as a protein substrate for PPIase_pro_ activity, because of the *cis-trans* isomerization of two peptidyl prolyl bonds (Tyr38-Pro39 and Ser54-Pro55) of RNase T_1_ are rate-limiting steps for its folding [45–47]. RNase T_1_ was first unfolded using guanidine hydrochloride. Refolding was then initiated by an 80-fold dilution in the presence or absence of the enzyme. The refolding reaction can be easily monitored by measuring the increase of tryptophan fluorescence.

**Figure 10.**
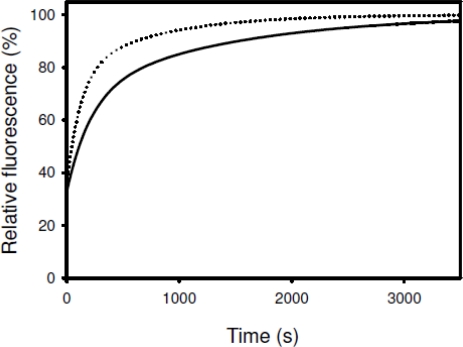
Catalysis of the slow refolding reactions of RNase T_1_ by SIB1 FKBP22 (PPIase_pro_ activity). The increase in tryptophan fluorescence at 323 nm during the refolding of RNase T_1_ (0.2 μM) is shown as a function of the refolding time. Refolding reactions were carried out at 10 °C in the absence (solid line) or presence of 8.9 nM of SIB1 FKBP22 (broken line). This figure was reproduced and modified from Suzuki *et al.* [26].

Acceleration of the two rate-limiting prolyl isomerizations was observed when refolding of RNase T_1_ is accompanied by the activities of SIB1 FKBP22 (Figure 10), suggesting that SIB1 FKBP22 catalyzes prolyl isomerization of proteins in a nonspecific manner. Temperature dependence of foldase activity was also examined by measuring activity at 10 and 20 °C [26]. The catalytic efficiency (*k_cat_*/*K*_m_) of SIB1 FKBP22 was greatly reduced at 20 °C (0.13 μM^−1^ s^−1^) as compared to that at 10 °C (0.50 μM^−1^ s^−1^).

Acceleration of this refolding reaction was also observed in the presence of *E. coli* FKBP22 [26]. However, it has been reported that the catalytic efficiency of *E. coli* FKBP22 increased when the temperature was increased to 20 °C. These results support previous results that found that the apparent optimum temperature of the PPIase_pep_ activity of SIB1 FKBP22 was 10 °C. It was also found that PPIase_pro_ activity was not detectable on single domain of FKBP (FKBP12) [48]. Meanwhile, multidomains of FKBPs (SlyD, FkpA, trigger factor, FKBP17, FKBP52) efficiently catalyzed folding of RNase T_1_ [17–23]. This evidence prompted us to propose that the presence of additional domains of SIB1 FKBP22, other than its catalytic domain, generates PPIase_pro_ activity.

**Figure 11.**
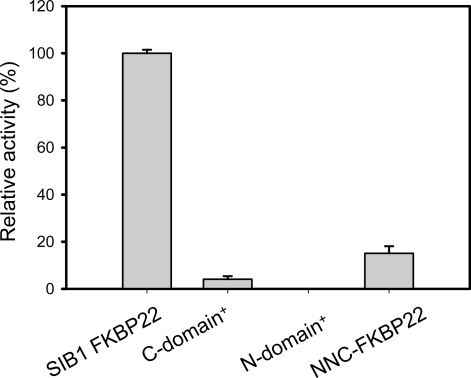
PPIase_pro_ activities of SIB1 FKBP22 and its derivatives measured at 10 °C. Relative PPIase_pro_ activities of the SIB1 FKBP22 derivatives, which are calculated by dividing the *k*_cat_/*K*_m_ value of the SIB1 FKBP22 derivatives by that of the wild-type protein (SIB1 FKBP22), are shown. The *k*_cat_/*K*_m_ value of WT was 0.5 μM^−1^ s^−1^. This figure was reproduced and modified from Suzuki *et al.* [39].

The importance of additional domains of SIB1 FKBP22 for PPIase_pro_ activity has been confirmed by characterization of PPIase_pro_ activity of the N-domain^+^, C-domain^+^, and NNC-FKBP22 [39,40]. As shown in Figure 11, the C-domain^+^, where the catalytic site is located, exhibited much less PPIase_pro_ activity compared to that of SIB1 FKBP22. In addition, PPIase_pro_ activity of the N-domain^+^ was abolished. Interestingly, NNC-FKBP22, which contains N- and C-domains, also exhibited much less PPIase_pro_ activity compared to that of SIB1 FKBP22. This result suggests that the presence of the C-domain with full length of α3-helix is a minimum requirement for PPIase_pro_ activity. The V-shaped structure of SIB1 FKBP22 is responsible for maximal PPIase_pro_ activity. Since the V-shaped structure is facilitated by dimerization of the N-domain, an additional N-domain is required for maximum PPIase_pro_ activity. Similar results have been also obtained from other multidomains of FKBPs, including *E. coli* FkpA and *E. coli* SlyD [19,48], in which removal of additional domains greatly reduces catalysis of protein substrates. In addition, the additional domain alone was unable to exhibit foldase activity.

### 5.2. SIB1 FKBP22 as a Chaperone

Chaperones are characterized by their ability to bind unfolded protein or molten globules and thereby prevent aggregation during folding. We then examined the chaperone activity of SIB1 FKBP22 by binding to a folding intermediate of protein. Reduced and carboxymethylated (RCM) α-lactalbumin has been used as a model for a folding intermediate of a substrate. This substrate has been commonly used to analyze chaperone activity of GroEL [49,50] and the FKBP family of proteins [51,52]. α-lactalbumin is stabilized by four disulfide bonds and a single Ca^2+^ ion [53,54] and therefore reduction of these disulfide bonds produces proteins with a partially folded molten globule-like structure [55]. This conformational change was confirmed by measuring far-UV CD spectra of this protein in the presence and absence of 2 mM DTT, which represents reduced and non-reduced proteins, respectively [44]. The spectrum of the reduced protein exhibited a trough with a minimum ellipticity value of −14,000 at 205 nm, accompanied by a shoulder with an ellipticity value of −7000 at 220 nm. The spectrum of the non-reduced protein, on the other hand, exhibited a broad trough with two minimum ellipticity values of −12,500 at 208 nm and −12,000 at 220 nm.

**Figure 12.**
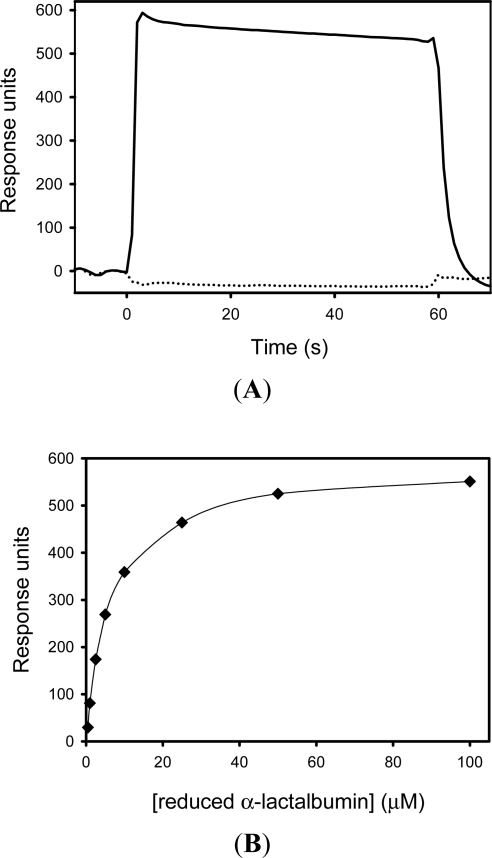
Binding of reduced α-lactalbumin to SIB1 FKBP22. (**A**) Sensorgrams from Biacore X showing the binding of reduced α-lactalbumin (100 μM) to immobilized SIB1 FKBP22 (solid line). The sensorgram showing the binding of non-reduced α-lactalbumin (100 μM) to SIB1 FKBP22, is also shown (broken line). Injections were made from time zero for 60 s. (**B**) Relationships between the equilibrium binding response and concentration of reduced α-lactalbumin. The equilibrium binding responses of SIB1 FKBP22 (closed diamond) is shown as a function of concentration of reduced α-lactalbumin. The solid line represents the fitting curve of a single binding site affinity model using the BIAevaluation program. These figures were reproduced from Budiman *et al.* [40], which is comparable to the result of Suzuki *et al.* [56].

The interaction between SIB1 FKBP22 with α-lactalbumin was monitored by surface plasmon resonance imaging using the Biacore X instrument (GE Health care, USA). Reduced and non-reduced α-lactalbumins (100 μM each) were injected onto the sensor chip, on which SIB1 FKBP22 was immobilized. An increase of resonance units (RU) was detected when reduced α-lactalbumin was injected, but was not detected when non-reduced α-lactalbumin was injected onto the same sensor chip (Figure 12). This result indicates that SIB1 FKBP22 does not bind to non-reduced α-lactalbumin, but will bind to reduced α-lactalbumin.

A *K*_D_ value for the binding of reduced α-lactalbumin to SIB1 FKBP22 was determined to be 2.2 μM [56], which is comparable to our latest report [40]. This value was determined by measuring equilibrium binding responses at various concentrations of reduced α-lactalbumin (Figure 12).

The ability of SIB1 FKBP22 to bind to a folding intermediate protein raised further questions regarding the binding site for the protein substrate in SIB1 FKBP22. To address this issue, we have analyzed the binding affinity of each domain (N- and C-domain^+^) and an engineered monomeric mutant, NNC-FKBP22, to a protein substrate. Results showed that the N-domain^+^ and NNC-FKBP22 do not bind to non-reduced α-lactalbumin, but will bind to reduced α-lactalbumin [40,56]. However, increasing RU values were not detected when non-reduced or reduced α-lactalbumin was injected onto a sensor chip, on which C-domain^+^ was immobilized, indicating that the C-domain^+^ does not bind to either non-reduced or reduced α-lactalbumin [56].

The binding affinity of the N-domain^+^ was comparable to that of SIB1 FKBP22 [56] (Table 2). By contrast, the *K*_D_ value for binding of reduced α-lactalbumin to NNC-FKBP22 was 6.5-fold higher than that of SIB1 FKBP22, indicating that the binding affinity of NNC-FKBP22 to a folding intermediate of proteins is greatly reduced as compared to that of SIB1 FKBP22.

Altogether, the binding site for the protein substrate is located at the N-domain, which is separated from the catalytic site of the C-domain. Our results showed that the binding site of SIB1 FKBP22 for a folding intermediate of protein is located at the N-terminal domain, which supports the proposal by Arie *et al.* [57] and Saul *et al.* [19] that the chaperone and PPIase activities of FkpA reside in the N- and C-terminal domains, respectively. The low affinity of NNC-FKBP22 indicated that a V-shaped structure of SIB1 FKBP22 is important for the efficient binding to a protein substrate [40]. This evidence may explain the low PPIase_pro_ activity of the C-domain^+^ and NNC-FKBP22. These mutants lack the V-shaped structure and are therefore unable to efficiently bind to the protein substrate (RNase T_1_). In addition, the N-domain^+^ is likely able to efficiently bind to RNase T_1_; however, PPIase_pro_ activity could not be detected due to the removal of the catalytic site.

However, the ability of SIB1 FKBP22 to prevent chemical or heat-induced aggregation remains to be investigated. Our latest study showed that SIB1 FKBP22 could effectively prevent DTT-induced aggregation of insulin, which indicated that SIB1 FKBP22 clearly exhibited chaperone activity. This information is now being submitted to be published elsewhere.

### 5.3. The Relationship between Foldase and Chaperone Activities

Chaperone activity is required for binding to a protein substrate. This binding is efficiently facilitated by the V-shaped structure through a “Mother’s arm” model, as proposed for the substrate binding mechanism of *E. coli* FkpA [58]. According to this model, two long α3 helices act as flexible “arms”, which can bend at the “elbows” (presumably located at the middle of the α3 helices). Two catalytic domains act as “hands” and the active site residues act as “fingers” for protein substrates. As a “mother” holds her “baby” by bending both of her arms, a dimeric form of *E. coli* FkpA holds a protein substrate by bending its two long α3 helices. A V-shaped structure of SIB1 FKBP22 may also be required to hold a protein substrate by a similar mechanism. The plasticity of the V-shaped structure may lead to a conformational flexibility to adopt various kinds of protein substrates. Once the protein is cradled by SIB1 FKBP22, the plasticity of the two α3 helices allows access to the isomerase’s catalytic site. The C-domains from each monomer of SIB1 FKBP22 attaches to the substrate and leads to the isomerization of the prolyl bonds. This idea explains the adaptability of the V-shaped structure to accelerate the folding of protein substrates through substrate binding and isomerization of the *trans-cis* prolyl bonds of the protein substrate.

## 6. A Possible Role of SIB1 FKBP22 in Cold Adaptation

SIB1 FKBP22 has been shown to be overproduced when the cells are grown at low temperatures and has shown typical features of cold-adapted enzymes. These features prompted us to propose that SIB1 FKBP22 is involved in cold adaptation of *Shewanella* sp. SIB1. However, it remains to be clarified exactly how FKBP22 is involved in the cold-adaptation of *Shewanella* sp. SIB1. According to the aforementioned results, we propose that SIB1 FKBP22 is important for folding proteins in the cells of *Shewanella* sp. SIB1 when grown at low temperatures. This might be due to the ability of SIB1 FKBP22 to act as a foldase and chaperone at low temperatures.

We believed that, as for other bacteria [1], exposing *Shewanella* sp. SIB1 to low temperatures causes a series of cellular problems, including the folding of proteins. In respect to the folding of proteins containing a *cis-*proline residue, *cis-trans* isomerization becomes much slower at low temperatures, since it is thermodynamically linked [11]. Moreover, slow folding, which might be caused by slow *cis-trans* isomerization, promotes others problems, which include the misfolding or degradation of protein substrates.

To address this problem, SIB1 FKBP22 was significantly increased when *Shewanella* sp. SIB1 was grown at low temperatures. This evidence was obviously shown by our 2D-PAGE analysis, as previously described. Once expressed, SIB1 FKBP22 will act as foldase through its PPIase_pro_ activity to catalyze a rate-limiting step of folding proteins containing a *cis*-proline residue. This response is part of strategy used for a cold adaptation process through proper folding of proteins and/or maintenance of the quaternary structure of proteins [59].

The ability of SIB1 FKBP22 to act as a foldase is supported by its chaperone activity that facilitates the binding affinity to a folding intermediate protein substrate. In the absence of chaperone activity, the PPIase_pro_ activity of SIB1 FKBP22 was significantly decreased and therefore could not act as an efficient foldase. Moreover, chaperone activity of SIB1 FKBP22 may also be important to prevent aggregation of proteins without a *cis*-proline residue. It has been reported that low temperature caused denaturation and aggregation of proteins [1]. However, the ability of SIB1 FKBP22 to prevent aggregation of proteins remains to be shown experimentally.

It is also possible for SIB1 FKBP22 to have other roles for the cold adaptation of *Shewanella* sp. SIB1. It has been reported that PPIase is involved in various biological processes including cell signaling, cell cycling, immune responses, and neural function [60]. However, the involvement of SIB1 FKBP22 in other biological functions of *Shewanella* sp. SIB1 and its relationship with cold adaptation remains to be clarified.

## 7. Other PPIases and Involvement in Cold Adaptation

Requirements of PPIases for cold adaptation have been observed in various organisms, regardless of whether they are hyperthermophiles, mesophiles or psychrophiles. These findings also revealed that all members of PPIases may be involved in cold adaptation with unclear mechanism.

Apart from SIB1 FKBP22, other FKBP members were also reported to be involved in cold adaptation in various organisms. FKBP18 from a hyperthermophilic archea, *Thermococcus* sp. KS-1, has been thought to be important at lower growth temperatures than the optimum in *Thermococcus* sp. KS-1 cells [61]. In the mesophilic bacterium, *E. coli,* the cold inducible trigger factor (*Ec*TF) has been reported to be important for cold adaptation of *E. coli* to low temperatures. Inactivation of the gene for *Ec*TF resulted in decreased viability of *E. coli* in cold temperatures [62]. Pyshcrophilic trigger factor of Antartic bacterium *Pseudoalteromonas haloplanktis* TAC125, *Ph*TF, was also reported to be a primary chaperone for growth in the cold [10]. It is interesting to note that these cytoplasmic proteins exhibit both PPIase (either toward peptide and protein substrates) and chaperone activities as indicated by the ability to prevent aggregation. It is reminiscent of the ability of SIB1 FKBP22 and therefore FKBP18, *Ec*TF and *Ph*TF may also be involved in cold adaptation through their foldase and chaperone activities.

Other PPIases that have been previously linked to low-temperature adaptation include cell-associated PpiB of *Bacillus subtilis* [24] and RotA of *Erwinia chrysanthemi* [63]. Based on the sequence similarities, these proteins belong to cyclophilin-like protein. However, there is no further information on the foldase and chaperone activities of these proteins. It is noted that cyclophilin is also considered as foldase based on its ability to accelerate the refolding of RNase T_1_ *in vitro*. Considering that PpiB and RotA are periplasmic proteins, it is suspected that these proteins are involved in cold adaptation, by facilitating protein folding in the periplasm, as other periplasmic PPIases do [64]. In addition, perisplasmic PPIase has also been reported to participate in the assembly of the outer membrane proteins [65]. However, it remains unclear whether this function has any relation to the cold adaptation mechanism.

*Cs*PinA, a parvulin member of psychrophilic archaeon *Cenarchaeum symbiosum*, is thought to have an important role in the survival of this archaeon at low temperature [25]. It is commonly known that parvulin exhibits foldase and chaperone activities, which seems to be also exhibited by *Cs*PinA. Both activities may be utilized for overcoming cellular problems that occur at low temperature. Interestingly, parvulin is unique in comparison to other PPIase members since parvulin prefers phosphorylated serine or threonine N-terminal to proline residue [60]. Since phosphorylated substrates are usually related to various biological processes (signal transduction, cell cycle, apoptosis), parvulin is believed to be involved in such biological processes in the cells [60]. However, experimental evidences are necessary to clarify the relationship between these biological roles with cold adaptation mechanism.

Noteworthy, it was found that some PPIases are secreted out of the cells, such as HP0175 of *Helicobacter pylori* and MIP of *L. pneumophila* [66,67]. But, these proteins have also been reported to be important for the growth of these organisms at low temperatures. Since these proteins are secreted out of the cell, the mechanism of their involvement in cold adaptation may differ to that of unsecreted PPIases, which are active inside the cell. *L. pneumophila* MIP is reported to contribute in facilitating protein secretion. *L. pnemumophila MIP* interacts with a newly secreted protein and, with its PPIase and/or chaperone activities, causes changes that convert the protein from enzymaticaly inactive to active prior or upon being secreted out the cell [67]. It is reported that the abundance of protein presents in wild-type culture supernatants changes upon the decrease in growth temperature of *L. pneumophila* [68]. Although there is no further information for the involvement of HPO175 in cold adaptation, it is speculated that this protein employs a similar mechanism with *L. pneumophila* MIP, as other secreted PPIase.

## 8. Conclusions

Previous studies have reported that PPIases are involved in cold adaptation of various organisms as part of their cold shock responses and adaptive mechanisms. However, there is no detailed explanation for the mechanism of involvement of PPIase in cold adaptation. In this review, we used SIB1 FKBP22 as a model protein, which was involved in the cold-shock responses of *Shewanella* sp. SIB1. Our extensive studies on SIB1 FKBP22 have revealed that this protein may be involved in cold adaptation through its ability to assist the folding of proteins at low temperatures, either as a foldase or a chaperone. As a foldase, SIB1 FKBP22 catalyzes the folding of proteins containing a *cis-*proline residue, where its folding rate is significantly restricted by *cis-trans* isomerizaton. As a chaperone, SIB1 FKBP22 was able to bind nonnative protein substrates, which included proteins containing *cis-*proline residues, during the folding reaction. Moreover, SIB1 FKBP22 may prevent the aggregation of proteins during folding through its chaperone activity. To our knowledge, SIB1 FKBP22 is the only PPIase from psychrophilic bacteria that has been extensively studied so far.

Apparently, foldase and chaperone activities are common mechanisms of PPIase which is involved in cold adaptation. However, additional mechanisms could be attributed for some periplasmic and secreted PPIases. Periplasmic PPIases are apparently not only involved in folding of various proteins in the periplasm, but also for assembling of outer membrane proteins. Meanwhile, secreted PPIases facilitate secretion of proteins that are necessary for the survival of microorganisms at low temperature. More extensive work to broaden our knowledge on these additional mechanisms of PPIases for cold adaptation is necessary.

## Figures and Tables

**Table 1.** Thermodynamic parameters for heat induced unfolding of the protein ^a^.

**Protein**	**Transition**	***T*_m_ (°C)**
SIB1 FKBP22	First	32.5
	Second	46.4
C-domain^+^		35.6
N-domain^+^		44.4

The melting temperatures (*T*_m_) of SIB1 FKBP22, N- and C-domains^+^ were deduced from the DSC curves as shown in Figure 9.

**Table 2.** The *K_D_* values of SIB1 FKBP22 and its derivatives. The *K_D_* values of SIB1 FKBP22, the N-domain^+^ and C-domain^+^ are reproduced from Suzuki *et al.* [56]. The *K_D_* value of NNC-FKBP22 is taken from Budiman *et al.* [40] and normalized on the basis of data from Suzuki *et al.* [56].

	***K_D_* Values (μM)**
SIB1 FKBP22	2.2
N-domain^+^	3.4
C-domain^+^	not detected
NNC-FKBP22	14.4
